# A systematic review of global publications on clouded leopard (*Neofelis nebulosa*): identifying the publication trends, research gaps, and future directions to strengthen its conservation

**DOI:** 10.7717/peerj.20421

**Published:** 2025-12-19

**Authors:** Nikita Phuyal, Nishan Kc, Neeta Pokharel, Shreejan Gautam, Nitu Adhikari, Bijaya Dhami, Saurav Lamichhane, Mahamad Sayab Miya, Abhinaya Pathak, Bijaya Neupane

**Affiliations:** 1 School of Forestry and Natural Resource Management, Tribhuvan University, Kathmandu, Bagmati, Nepal; 2 WWF Nepal, Baluwatar, Kathmandu, Bagmati, Nepal; 3 Institute of Forestry, Pokhara Campus, Tribhuvan University, Pokhara, Gandaki, Nepal; 4 Department of Biological Sciences, University of Alberta, Edmonton, Alberta, Canada; 5 Research Centre for Terrestrial Ecosystem Science and Sustainability, Harry Butler Institute, Murdoch University, Perth, Australia; 6 Department of Biological Sciences, Western Kentucky University, Bowling Green, KY, United States of America; 7 Department of Biological Sciences, University of California, San Diego, California, United States of America; 8 Department of Forest Sciences, Faculty of Agriculture and Forestry, University of Helsinki, Helsinki, Finland

**Keywords:** Clouded leopard, Conservation biology, Habitat use, Population dynamics, Molecular ecology, Wildlife threats, Human-wildlife conflict, Biodiversity conservation, Research gaps, Protected areas

## Abstract

**Background:**

Despite global investment in studying, protecting, and managing carnivores, species like the clouded leopard *Neofelis nebulosa* (Griffith, 1821), renowned for its elusive nature, remain significantly understudied. There is also insufficient knowledge of clouded leopard research trends in spatial and temporal domains. Additionally, thematic areas of research on this species are not clearly known. This gap in information may hinder the development of effective strategies to address key conservation challenges such as habitat loss, poaching, and illegal trade.

**Methods:**

To bridge these gaps, we systematically reviewed 123 peer-reviewed journal articles published up to December 2022, offering critical insights into the current state of knowledge and identifying future research priorities to inform conservation planning.

**Results:**

The spatial analysis of clouded leopard research reveals that Thailand (*n* = 28) dominates the range countries, while the USA (*n* = 26) dominates non-range countries in terms of research efforts. Temporally, research output has shown a significant increase since 2006, peaking in 2016 (*n* = 13), with a positive trend in publications (Kendall’s tau = 0.52, *P* < 0.001). Most studies focused on anatomy and physiology in captive populations (*n* = 31) and habitat use and distribution in free-ranging populations (*n* = 23). The studies on the impact of climate change on the clouded leopard and its habitat, alongside feeding ecology, remain scant, necessitating the future research in these areas. Our analysis also revealed that the maximum number of publications employed diagnosis and treatment (26%), followed by camera trapping (24.4%). We recommend integrating local ecological knowledge and monitoring technologies to map the clouded leopard’s corridors, connectivity, and bottleneck sites at the landscape level. A higher number of publications addressed habitat loss and illegal trade as the primary threats to clouded leopard conservation. Effective law enforcement, proper land use, land cover planning, and community engagement are crucial for conserving this species. Moreover, clouded leopard range countries are recommended to develop sustainable financial mechanisms and implement the conservation action plan across the country, which can improve conservation outcomes.

## Introduction

The clouded leopard is a medium-sized predator of the Felidae family distributed to the South and Southeast Asia. Although classically considered a single species, it has two species: *Neofelis nebulosa*, which is limited to the mainland of Southeast Asia, and *N. diardi*, which is restricted to the islands of Borneo and Sumatra (Buckley-Beason et al., 2006; Kitchener, Beaumont & Richardson, 2006; Wilting et al., 2007). Clouded leopard is listed under the vulnerable category of the IUCN (Gray et al., 2021) and Appendix I of the CITES. Besides, in most of its home range, it is protected by national legislation (D’Cruze & Macdonald, 2015). There are fewer than 3,700–5,580 mature individuals of clouded leopard throughout its range (Gray et al., 2021). Its density varies between 0.3 and 5.14 mature individuals per 100 km^2^ in the protected areas of the Indian subcontinent, northern Myanmar, Malaysia, and Thailand (Borah et al., 2013; Petersen, Savini & Ngoprasert, 2020).

The clouded leopard is a unique species characterized by distinguished physical features, reproductive traits, and diet. It has a spotted head with two broad bars on its neck and stripes on its cheek. It has a warm ochre coat with grey elliptical clouds with black edges, which turn into blurred rings on its long tail and into black oval spots on the legs (Jnawali et al., 2011). Its canines are remarkably elongated (Sunquist & Sunquist, 2002). It becomes sexually mature at the age of about two years and produces 1–5 cubs after a gestation period of 85–93 days (Jnawali et al., 2011). The female weighs between 11.5 and 13.5 kg, and the male weighs between 16 and 18 kg (Austin & Tewes, 1999; Grassman et al., 2005). It feeds mainly on small mammals such as hog deer, wild pig, rodents, slow loris, porcupines, squirrels, and pangolins (Grassman et al., 2005; Mohamad et al., 2015; Rasphone et al., 2022) and birds such as pheasant (Nowell & Jackson, 1996).

The clouded leopard is found in a wide range of habitats, including different forest covers and elevational ranges. It is a nocturnal and highly arboreal species found primarily in evergreen tropical rainforests, dry and deciduous forests, and logged forests (Gray et al., 2021). However, it is sometimes found in grassland, scrubs, dry tropical forests, as well as mangrove swamps (Nowell & Jackson, 1996). It prefers forests over open habitats. The home range for males and females is similar, which is between 30 and 40 km^2^ (Grassman et al., 2005; Austin et al., 2007) and found within an elevation range of 0–3,500 m (Can et al., 2020). However, for any habitat range, its density increases with the decreased density of cohabiting larger cats such as the tiger (*Panthera tigris tigris*) and leopard (*Panthera pardus*) (Lynam, 2001; Petersen, Savini & Ngoprasert, 2020).

The clouded leopard is widely distributed in protected and fragmented habitats across home range countries (Gray et al., 2021). In China, the species was previously assumed to be restricted to the Yunnan Province and the Tibetan Autonomous Region (Ma et al., 2022). Recent studies have advanced our understanding of its range, with Ding et al. (2025) documenting new records in Qomolangma National Nature Reserve, Tibet, ∼1,000 km beyond prior known Chinese populations, suggesting novel dispersal corridors, and Wang et al. (2025) identifying ∼38% of Taiwan as suitable for reintroduction despite historical extirpation. Its record is confirmed from Chitwan, Parsa, Langtang, Makalu Barun, and Shivapuri Nagarjuna national parks and Annapurna, Kanchenjunga, and Manaslu conservation areas of Nepal (Ghimire, 2010; Jnawali et al., 2011; Ghimirey et al., 2012; Ghimirey et al., 2013; Pandey, 2012; Ghimirey, Acharya & Dahal, 2014; Lamichhane et al., 2014; Can et al., 2020). In Bhutan, it is probably found in the southern forested areas in both protected and outside protected areas (Penjor et al., 2018). It is widely found in the northeastern part to the west in the Valmiki Tiger Reserve, Sikkim, West Bengal, Assam, Arunachal, Meghalaya, and Mizoram of India (Bashir et al., 2011; Borah et al., 2013; Shafi, Maurya & Gupta, 2019; Mukherjee et al., 2019; Jhala, Qureshi & Yadav, 2020). In Bangladesh, the species was photographed in the Sangu-Matamuhuri Reserve Forest (Alliance, 2016). In Myanmar, it is distributed to the northern region (Kachin and Sagain) and southern region (Kayin and Taninthayi) (Zaw et al., 2014; Greenspan et al., 2020). In the Lao People’s Democratic Republic (LPDR), the species remains in Nam Et- Phou Louey National Park, though it was previously found throughout the country (Rasphone, 2018). In Thailand, it is widely found throughout the country’s fragmented forests (Petersen, Savini & Ngoprasert, 2020). In Cambodia, the species is now recorded from the northeast (Siem Pang Wildlife Sanctuary and Virachey National Park) and southwest (Cardamom Rainforest Landscape) (Gray et al., 2017; McCann, Pawlowski & Soukhon, 2020). In Vietnam, no recent record is reported, though it was widespread throughout the evergreen forests (Willcox et al., 2014). In Malaysia, it is widely found across the Peninsular region (Gray et al., 2021).

The population of clouded leopard is declining in most of the range countries with reduced abundance and distribution. It was found that there was a 32.5% decline in its abundance between 1999 and 2019 (Gray et al., 2021). Its number is very low and close to extinction in Vietnam (Willcox et al., 2014), China, and Bangladesh, while it is already extinct on the island of Taiwan (Chiang et al., 2015). The species is mainly threatened by illegal hunting, trade (for skins, furs, and bones), incidental mortality due to snares set for other species, and habitat loss (Pacifici et al., 2013; Harrington, 2015; Petersen, Savini & Ngoprasert, 2020; Ghimirey & Acharya, 2020). In addition, documented challenges to the survival of clouded leopards include deforestation, fragmentation leading to reduced functional connectivity, and increasing infrastructure development within their habitats (Stibig et al., 2014; Ghimirey & Acharya, 2018; Kaszta et al., 2020). Recent advancements highlight further challenges: clouded leopard exhibit low genetic diversity, population-specific adaptations, and high inbreeding, highlighting the urgent need to incorporate evolutionary and genomic considerations into conservation planning (Yuan et al., 2023). Likewise, Sethi, Chauhan & Singh (2023) reported declining captive breeding success and imbalanced demographics in Indian zoos, underlining gaps in ex situ management. Similarly, climate-driven habitat and connectivity models project declines in suitable habitat and corridors under future climate change, highlighting the need for proactive translocation and reintroduction planning (Abedin et al., 2024; Can et al., 2020). Also, prey depletion could be another threat; however, further research is required to validate this fact (Gray et al., 2021).

Due to its elusive nature and limited studies on clouded leopard across its range countries, it is considered one of the least understood carnivore species (Buckley-Beason et al., 2006; Gray et al., 2021). The clouded leopard continues to receive less attention in terms of funding and research priorities compared to other large carnivores. This underscores the critical knowledge gaps in understanding their ecology, population dynamics, and human threats to them within their global ranges (D’Cruze & Macdonald, 2015). These information are crucial in developing effective conservation strategies for the species. Similarly, limited documented information on research trends and themes on clouded leopard research have further hindered the fostering of suitable strategies in conservation efforts.

Within this study, we aim to identify the current focus of clouded leopard research on the global scale from 1965 to 2022 (over six decades) and identify the key knowledge gaps to pave the way for an effective research and conservation roadmap. Addressing these gaps through a systematic review can provide a finer insight into the global status of clouded leopard research trends, its thematic focus, underrepresented areas, and spatial and temporal research patterns. We hypothesize that the trends in clouded leopard research in different thematic areas have insufficiently addressed the increasing conservation threats. For this, we systematically reviewed and analyzed the documented research on the clouded leopard from 1965 to 2022. First, we evaluated the spatial distribution of clouded leopard research across the range and non-range countries and analyzed the temporal trend of clouded leopard research globally. Second, we categorized research on different thematic areas and aligned their priorities for addressing conservation threats. Third, we evaluated the research approaches employed across different studies. Last, we synthesized the threats documented across the studies and the corresponding conservation recommendations proposed to address them. Such information can be helpful for wildlife managers and policymakers, which will assist to establish research and conservation priorities for protecting this threatened species at local, national, and transboundary levels. The output will also direct future researchers to focus on critical thematic areas currently lacking information for the conservation of this species.

## Materials & Methods

### Guideline

We systematically reviewed all the relevant literature on clouded leopard under the Preferred Reporting Items for Systematic Reviews and Meta-Analyses (PRISMA) guideline (Moher et al., 2009; Karki et al., 2021; Bist et al., 2021).

### Search strategies

All the published literature on clouded leopards was intensively searched using two electronic databases, Google Scholar and PubMed, available before December 31, 2022. Literature was searched by N.P. and N.K. using the advanced search box with a combination of predetermined keywords and the Boolean operator (OR). The combination of keywords includes “Clouded leopard” OR “*Neofelis nebulosa*” OR “*Pardofelis nebulosa* “OR “Formosan clouded leopard”. As another source, we used the Clouded Leopard Working Group’s (CLWG) literature resources to collate published literature.

### Selection criteria

Our study included the original research article and review articles (using secondary sources of information), primarily focused on clouded leopard. We retained articles on both free-ranging and captive settings of clouded leopard. Articles published in languages other than English were excluded from consideration. Additionally, grey literature, which comprises unpublished forms such as conference proceedings, dissertations, reports, book chapters, and preprints, was not included. There were no disagreements on classification. The articles published in predatory journals were excluded as they failed to adhere to publishing protocols and lacked a rigorous peer review process (Beall, 2016). To ensure the quality and reliability of the data, only those articles with full-text availability were considered for the final systematic review. In instances where the full text was not accessible through the initial database search, efforts were made to contact the corresponding authors and track the full texts through platforms such as Research Gate, which are well-known social networking sites for scientists and researchers.

### Article screening

**Figure 1:**
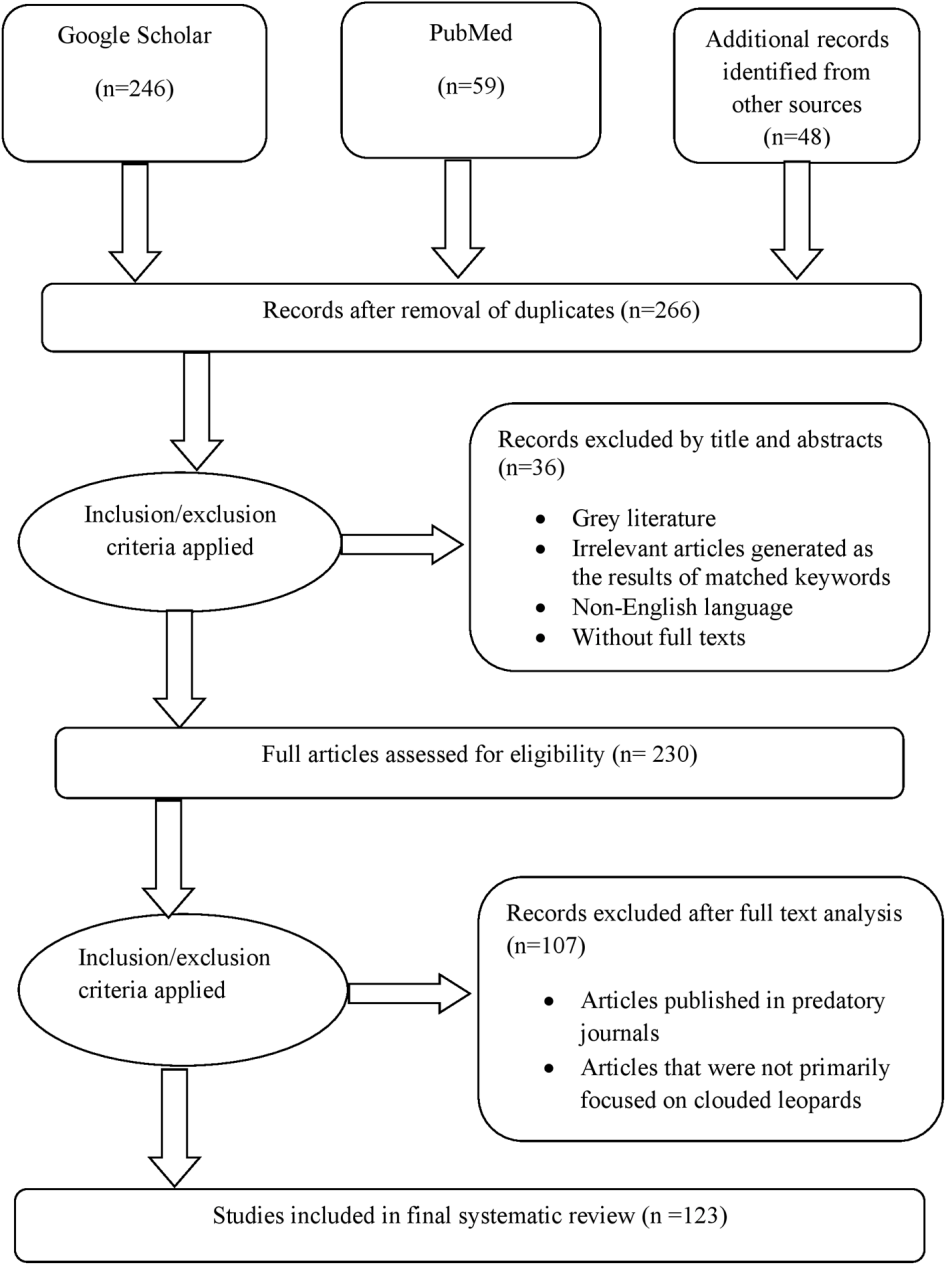
PRISMA (Preferred Reporting Items for Systematic Review and Meta-Analysis) flow diagram of literature search and articles selection process, adopted from Moher et al. (2009).

The first step (database search and additional source records) generated 353 articles (Fig. 1). Following the removal of duplicate articles, a refined set of 266 articles remained. N. Phuyal, N. Pokhrel, and N. Adhikari screened the resulting records. Screening titles and abstracts led to the exclusion of 36 articles, and the remaining 230 articles were assessed for further eligibility evaluation. Subsequently, the full text of those 230 articles was reviewed to determine their alignment with the review’s objectives (see details in Appendix S1). After analyzing the full texts, it was found that 123 articles met the criteria for inclusion in a detailed review. The literature search results and the selection process are summarized in Fig. 1.

### Data abstraction and analysis

We employed Microsoft Excel (Professional Plus Version 2013) to develop a structured data abstraction spreadsheet. The spreadsheet allowed for the extraction of five key elements of information from each article that was retrieved. These elements included: (a) the country where the study was conducted (b) the year of publication of the article (c) the study themes (d) the research approach (e) threats and recommendations addressed by the article.

We prepared eleven broad themes for captive and free-ranging studies to analyze the thematic focus of the publication and global research gap across both range and non-range countries (Table 1). The classification of themes was adopted from Sharma & Singh (2020) and Karki et al. (2021). Moreover, eight different research approach categories were prepared to pinpoint the field techniques utilized by each publication. The threats and recommendations addressed by each publication were classified based on a similar categorization from Bist et al. (2021). The statistical association between the number of publications and year of publication was investigated through the non-parametric Kendall’s tau correlation test using RStudio Version 2022.07.1 + 554 (RStudio Team, 2022).

**Table 1:** Thematic areas of the studies on clouded leopards and a summary of key contents are included in each thematic area.

**S.N.**	**Thematic area**	**Key contents**
1	Diet and food dynamics	Scats analysis, prey-predator interaction/relations, feeding science, prey selections, predation, foraging, food habits, prey availability
2	Conservation	Conservation management/strategies, captive breeding, action plan/strategies, ecological economics, identification of important conservation areas, species reintroduction, conservation implications
3	Habitat use and distribution	Habitat selection, occupancy survey, distribution surveys, modeling, spatial–temporal overlaps, first record/verification, distribution update, photographic record, co-existence with other predators, habitat suitability analysis
4	Human dimensions of conservation	People’s attitudes towards conservation of clouded leopard, subjective well-being, personality assessments
5	Review/synthesis	Review of clouded leopard ecology or other aspects
6	Threats	Factors affecting clouded leopard survival, anthropogenic activities, drivers of habitat loss and fragmentation, illegal trade of clouded leopard, the impact of socio-economic development on clouded leopard and its habitat
7	Movement ecology	Home range/size, movement patterns
8	Ethology	General behavior, social behavior, ethogram, activity budget pattern, reproductive behavior/success
9	Population	Population dynamics, site abundance, population density
10	Anatomy and physiology	Capture and chemical immobilization, reproductive physiology, hormones, diagnosis and clinical treatments, disease, parasites and pathogens
11	Molecular ecology	Phylogeography, genetic structure, genomes, genetic variation, phylogenetic analysis, genetic connectivity, genetic drift, genetic diversity, evolutionary differences, neotype

## Results

### Spatial publication trend on the clouded leopard

**Figure 2:**
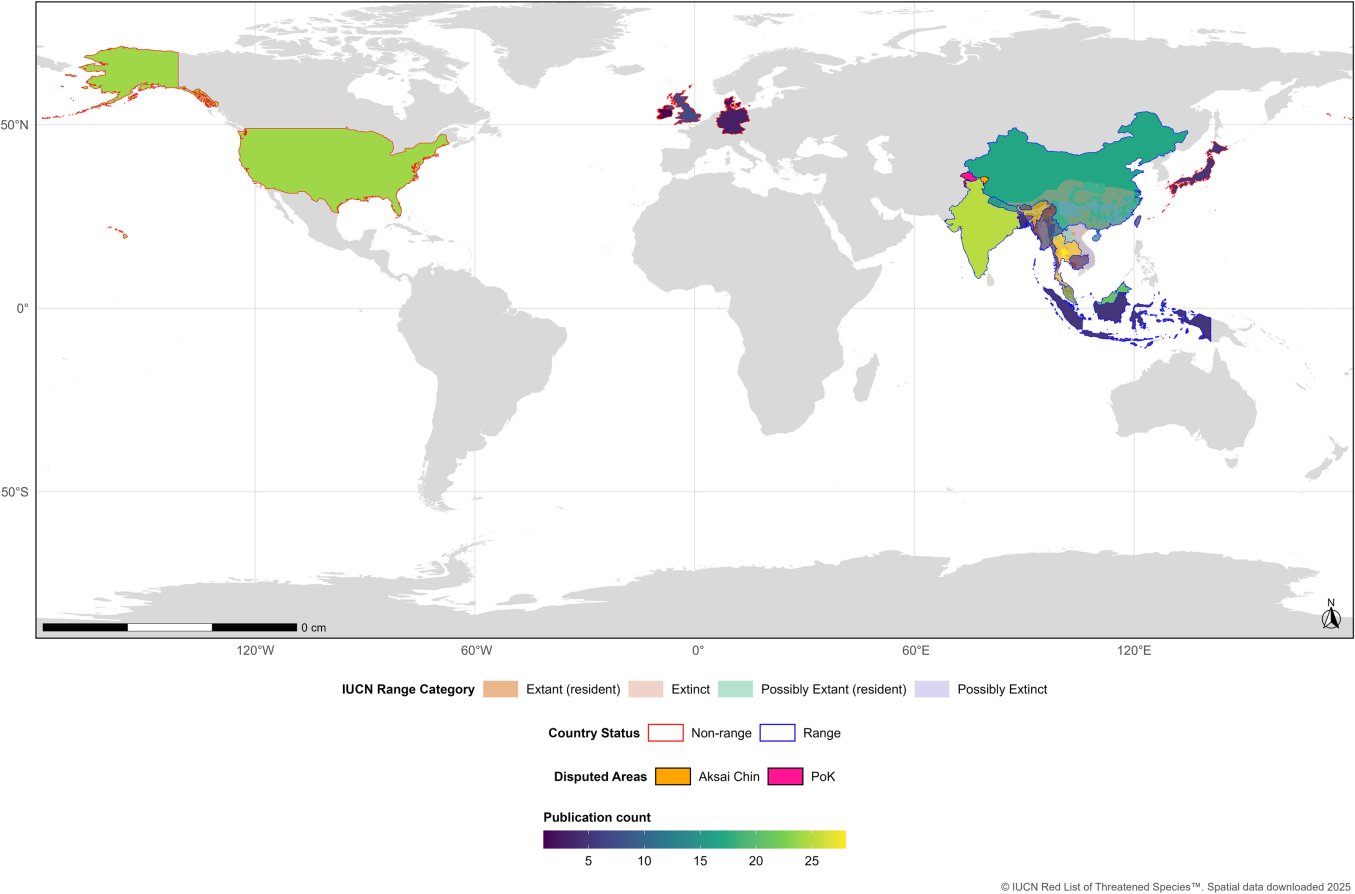
Spatial distribution of clouded leopard research across range and non-range countries.

The highest number of studies on clouded leopard were conducted in Thailand (*n* = 28), followed by India, China (*n* = 25) and the USA (*n* = 24). Among the range countries, Thailand (*n* = 28), India (*n* = 25), China (*n* = 25), and Malaysia (*n* = 21) dominated clouded leopard research, whereas Vietnam (*n* = 2), Bangladesh (*n* = 5), Cambodia (*n* = 5), and Laos (*n* = 6) were underrepresented. In contrast, among the non-range countries, most publications originated from the USA (*n* = 24), followed by the UK (*n* = 7), while Ireland (*n* = 1) and Denmark (*n* = 2) had the fewest (Fig. 2; Table 2; Table 3; Appendix S2).

When the publications were based on multiple countries, we allocated the publication number to each of the countries studied. Therefore, the total number of publications assessed in the current review study was not equal to the total number of publications of all the countries in the range.

### Temporal (yearly) publication trends on the clouded leopard

**Figure 3:**
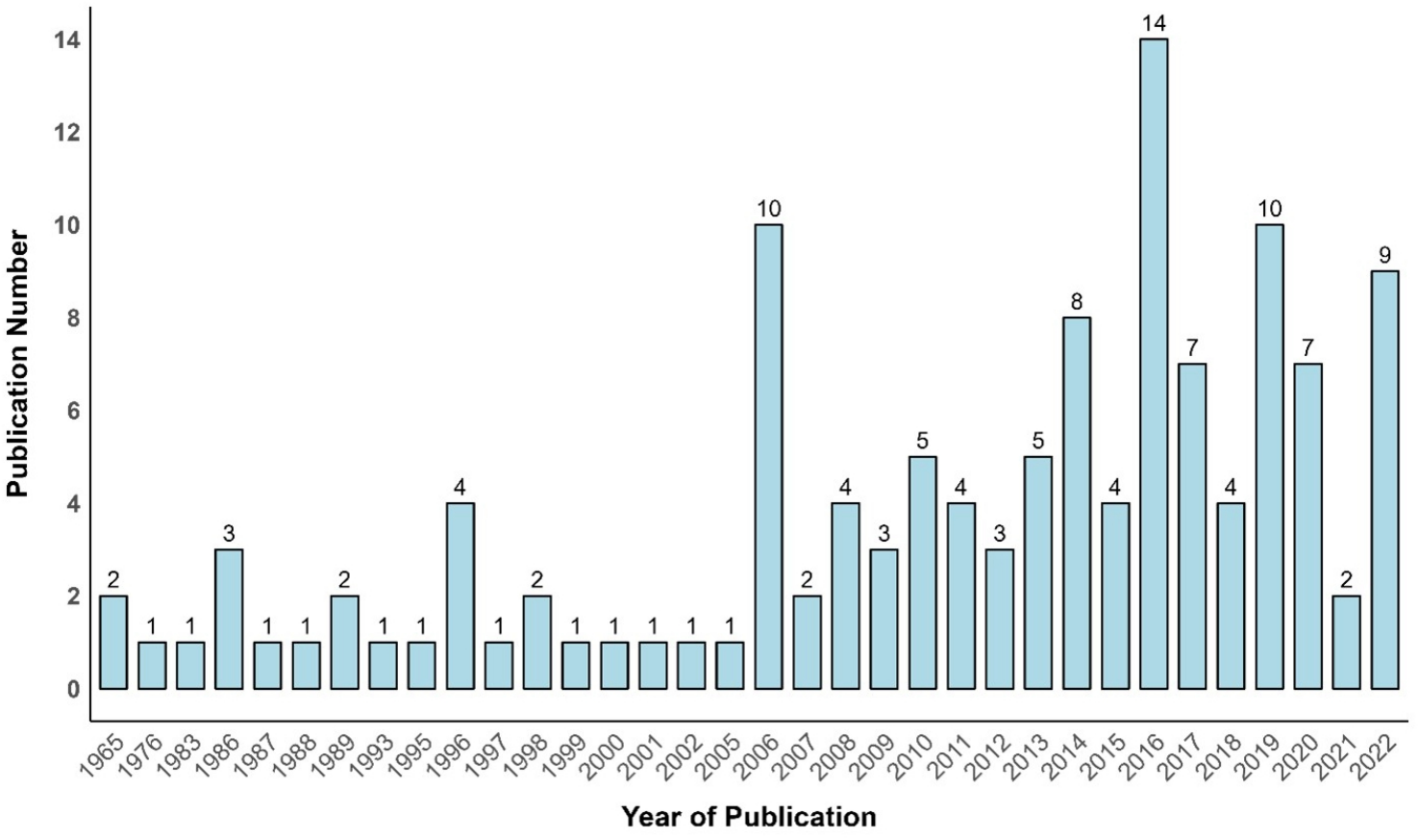
Yearly publication on clouded leopard between 1965 to 2022.

Publications on clouded leopard began in 1965, and only two articles were published during that particular decade (1965–1975). According to Kendall’s tau correlation test, the global publication trend revealed a significant positive increase in published research articles on clouded leopard from 2006 to 2022 (Kendall’s tau = 0.52, *P* < 0.001). However, the number of publications on clouded leopard gained momentum in 2016 (*n* = 13), followed by 2019 (*n* = 10), 2006 (*n* = 9), and 2022 (*n* = 9) (Fig. 3).

### Thematic focus

**Figure 4:**
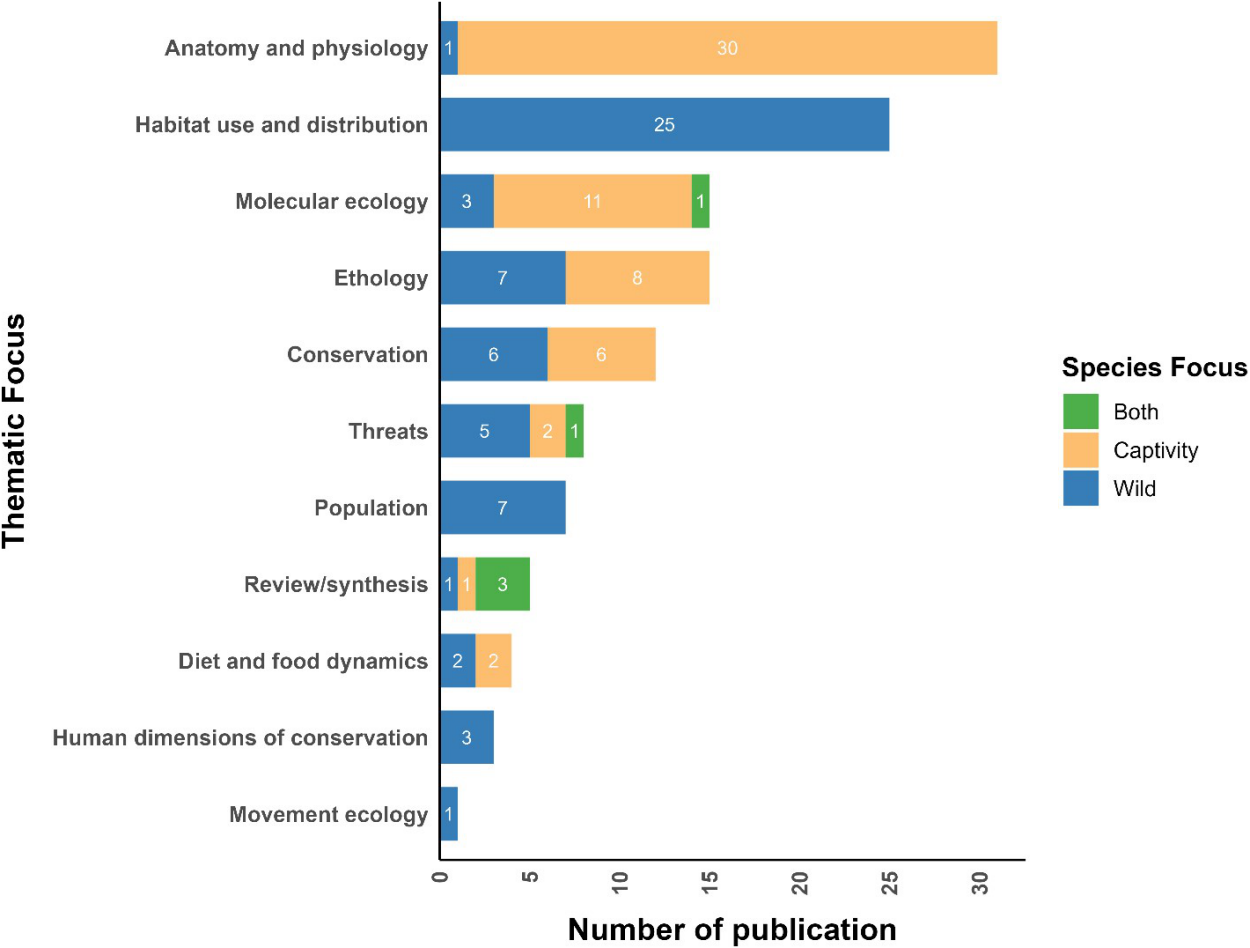
Thematic focus of the global publication on clouded leopard from 1965 to 2022.

Most of the research was conducted on a captive population (*n* = 67), while 56 publications were based on a free-ranging population. Of the eleven themes of clouded leopard research considered, the majority of the studies focused on anatomy and physiology (*n* = 31), followed by molecular ecology (*n* = 15) and ethology (*n* = 8) for the captive populations (Figs. 3 and 4). However, in the studies on free-ranging populations, the highest number of research studies focused on habitat use and distribution (*n* = 23), followed by population (*n* = 7) and ethology (*n* = 6). The least studied theme of clouded leopard in the captive populations was review/synthesis (*n* = 1). Similarly, movement ecology was the least studied theme for free-ranging populations (*n* = 1) (Figs. 3 and 4).

**Table 2:** Range countries of clouded leopard with publications from 1965 to 2022.

**Range countries of clouded leopard**	**Number of publications**
Bhutan	12
China	25
Bangladesh	5
Cambodia	5
India	25
Laos	6
Malaysia	21
Myanmar	8
Nepal	16
Thailand	28
Vietnam	2
Total	153

**Table 3:** The number of studies on clouded leopard for non-range countries.

**Non-range countries of clouded leopard**	**Number of publications**
Denmark	2
Germany	3
Indonesia	5
Ireland	1
Japan	5
UK	7
USA	24
Total	47

### Research approach

**Figure 5:**
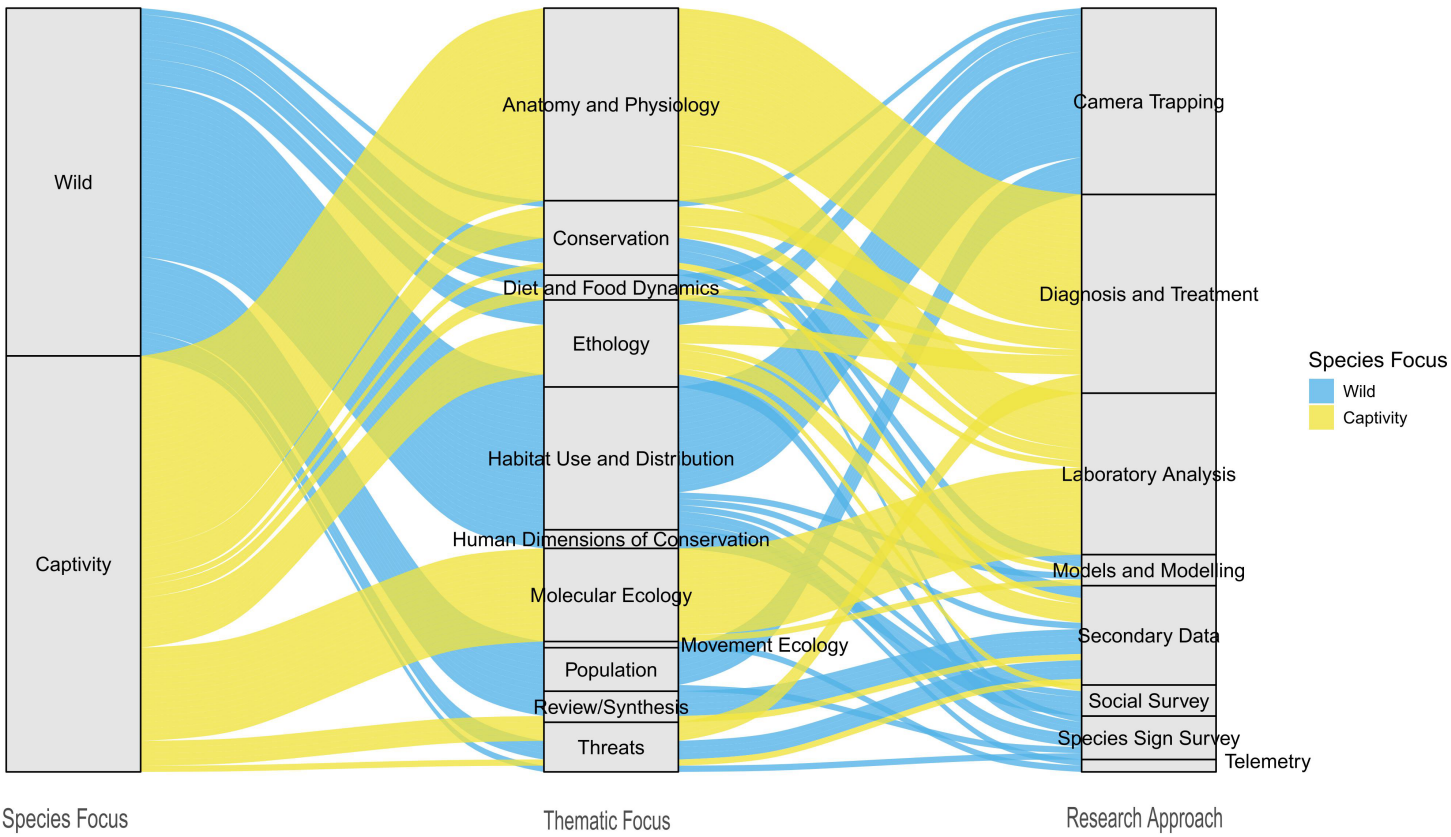
Sankey diagram showing the distribution of published studies on clouded leopard based on the population focus (wild *vs.* captivity), thematic focus, and employed research approaches.

**Figure 6:**
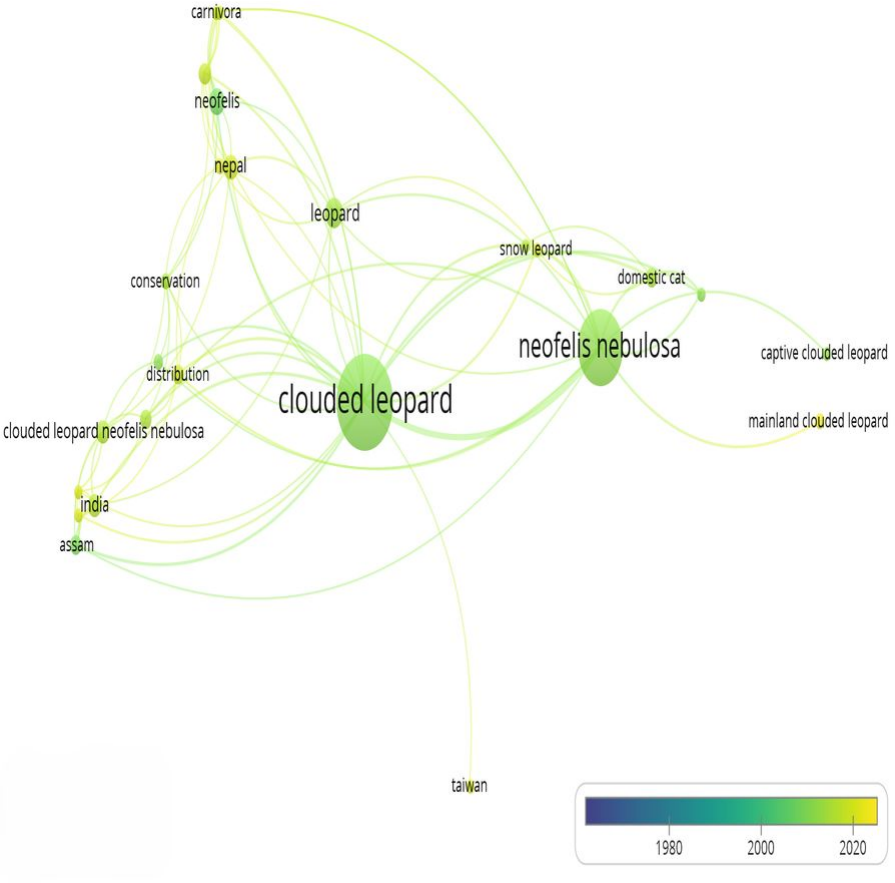
VOSviewer co-occurrence network of terms extracted from the title field of the published studies on clouded leopard, showing term clustering and interconnections based on co-occurrence frequency.

Research approaches were categorized into eight different topics: Camera Trapping, Diagnosis and Treatment, Laboratory analysis, Models and modeling, Secondary data, Social survey, Species sign survey, and Telemetry. Our analysis of the published articles on clouded leopard revealed that the maximum number of publications employed diagnosis and treatment (26%), followed by camera trapping (24.4%), laboratory analysis (21.1%), and secondary data (13%). By contrast, the least used research approach included species sign survey (5.69%), models and modeling (4.07%), social survey (4.07%), and telemetry (1.63%), indicating a notable gap in these methodologies within clouded leopard research (Fig. 5). Moreover, the co-occurrence network diagram highlights clouded leopard and *Neofelis nebulosa* as central keywords, with strong links to geographic regions (India, Nepal, Assam and Taiwan) and themes such as distribution and conservation (Fig. 6).

### Threats addressed by each publication

The threats mentioned in each publication were studied meticulously. Only one-third of the publications out of 123 mentioned threats faced by the vulnerable clouded leopard. The most prominent threat was habitat loss and fragmentation (*n* = 32). This was followed by illegal trade and poaching (*n* = 13) and diseases (*n* = 4) (see details in Appendix S3).

### Recommendations resulting from publications

Only 65 publications out of 123 recommended some measures for further studies and conservation of clouded leopard. The publications analyzed for this research made recommendations regarding future actions. Most of these are related to further research and monitoring (*n* = 43), signifying fewer studies in this species. It was followed by the need for better conservation and policies (*n* = 21), indicating an imminent need to save the remaining population of clouded leopard, habitat management (*n* = 6), and conservation education and awareness (*n* = 3) (Appendix S3).

## Discussion

### Spatial publication trend on the clouded leopard

The distribution of research efforts on clouded leopard varies significantly across regions, as analyzed through our systematic review of global publications. Most publications were available from Southeast Asia nations. Thailand is the leading contributor to clouded leopard studies, followed closely by India. Countries with more endangered species and a higher percentage of protected species tend to have more conservation and ecology articles in all scientific publications (Doi & Takahara, 2016). This correlation underscores the importance of conservation efforts in regions with rich biodiversity. For instance, clouded leopard, as studied by Petersen, Savini & Ngoprasert (2020), is widely distributed throughout Thailand’s fragmented forests, with camera trap records confirming their presence in major forest complexes (Grassman et al., 2005; Grassman et al., 2016). Similarly, clouded leopard is found in parts of eastern and northeastern India, including Arunachal Pradesh, Meghalaya, and Mizoram (Mukherjee et al., 2019), as well as in West Bengal and the Valmiki Tiger Reserve in Bihar (Shafi, Maurya & Gupta, 2019). The first camera-trap photographic confirmation of the clouded leopard in the Sikkim Himalaya by Sathyakumar et al. (2011) marked an important milestone in India’s research history, which expanded the known range of the species in the Indian Himalayas and contributed to filling a key geographical knowledge gap in its distribution. These findings collectively emphasize the need for continued research and conservation measures in these regions. Research efforts in other range countries such as China, Malaysia, Nepal, and Bhutan also indicate a degree of regional interest. However, as Gray et al. (2021) emphasize, even in these countries, along with India, Malaysia, and Thailand, the actual population trends of this elusive felid remain inadequately documented. This discrepancy suggests that while publication counts indicate some research activity, significant gaps persist in our understanding of the clouded leopard population.

National funding and political support often influence the extent and focus of conservation research (Vinkler, 2008; Doi & Takahara, 2016). Despite their critical roles in the clouded leopard’s range, the underrepresentation of Vietnam, Myanmar, and Laos suggests potential gaps in research funding, capacity-building, or collaborative networks in these countries. The vast majority of Myanmar is ecologically suitable for and within the historic range of the clouded leopard (Grassman et al., 2016). Petersen, Savini & Ngoprasert (2020) suggest that despite available habitat, clouded leopard is likely extirpated from all of Vietnam, most of China, and large parts of Laos and Cambodia, which is consistent with earlier studies (Willcox et al., 2014; Grassman et al., 2016). Populations of clouded leopard have decreased by over 30% between 1999 and 2019 across Myanmar, Cambodia, Laos, Vietnam, Bangladesh, and China, with these countries likely having higher populations in the past (Abedin et al., 2024). From these findings, it can also be inferred that the declining population trends of clouded leopard may be linked to the extent of research conducted by countries. The amount of research done in a particular area can impact how conservation is carried out locally and globally, as demonstrated by Doi & Takahara (2016). We recommend prioritizing targeted investments in funding, capacity-building, and international collaboration, particularly in underrepresented countries like Vietnam, Myanmar, and Laos, to develop and implement more effective, regionally tailored conservation strategies. The engagement of non-range countries like the USA and the UK underscores the global importance of conservation efforts. While their contributions are valuable for the conservation of the species, they also raise questions about the extent of collaboration with the range countries. The rise in publications from non-range countries may also reflect growing global concern over biodiversity loss and climate change (Doi & Takahara, 2016).

### Temporal (yearly) publication trends on clouded leopard

Our analysis indicates an increasing trend in the number of papers accessible online over the past three decades, with the highest number of papers published in 2016. This trend may be linked to increased awareness following the species’ listing as vulnerable by the IUCN and international trade regulations through CITES. The classification of the clouded leopard as vulnerable by the IUCN in 1986, coupled with the regulation of its trade internationally through CITES since 1975, might contribute to the rising awareness and interest in clouded leopard research. Similarly, the involvement of major international organizations such as the World Wildlife Fund (WWF), World Animal Protection, Wildlife Conservation Research Unit, and the Felidae Conservation Fund may have contributed to the growing trend of clouded leopard research.

### Thematic focus of clouded leopard research

The higher amount of research on captive populations suggests a strong interest in understanding the health and well-being of these animals in captivity. Researching the anatomy and physiology of free-ranging animals is challenging and costly. Moreover, adhering to standard ethical protocols is necessary during handling and sample collection procedures (Soulsbury et al., 2020). Minimizing total processing time is crucial to prevent stress, injury, or mortality (Ponjoan et al., 2008; Deguchi, Suryan & Ozaki, 2014), a particularly challenging task for free-ranging animals. These challenges likely explain why most anatomy and physiology studies on clouded leopards are based on captive individuals. The higher amount of research focusing on the habitat use and distribution of free-ranging clouded leopard populations reflects a strong interest within the scientific community in understanding habitat dynamics and the ecological requirements of the species. In this review, a cross-country comparison between range and non-range contexts was not feasible based on the available literature. This highlights the need for future research to better understand potential differences in habitat use across range countries’ contexts. Notably, most of these publications do not involve any procedures requiring direct contact with the species. This approach effectively addresses ethical challenges that may arise during research activities and is less time-consuming than research involving laboratory work or direct interaction with animals. Similarly, the use of motion-triggered cameras for long-term monitoring and activity of the species (Gil-Sánchez et al., 2011; Chiang & Allen, 2017), as well as the use of technology like Maxent (Phillips, Anderson & Schapire, 2006), GIS, and remote sensing data using bioclimatic as well as environmental variables (Macdonald et al., 2019; Shrestha et al., 2021), also make it easier to conduct research related to habitat use and distribution of wildlife species, contributing to the increased number of publications in this theme. Though the highest number of publications for free-ranging clouded leopard focus on habitat use and distribution, there are still gaps in research in areas such as home range and movement, activity patterns (but see Can et al. (2020)), interaction with other carnivores, and behavioral and communication studies of clouded leopard. Compared to species like *P. pardus*, the ecology and behavior of clouded leopard are relatively less understood and studied (Chiang & Allen, 2017). The issues of climate change and its threats to carnivores are pervasive (Leão et al., 2023). For the clouded leopard, climate change impacts documentation is observed at almost 4,000 m (Can et al., 2020). However, there are no empirical studies on the impact of climate change on clouded leopards and their habitat yet. Given that climate change can potentially alter the biogeographical characteristics of their existing range, understanding its implications is crucial for the long-term survival of this species (Zanin, Palomares & Albernaz, 2021). Studies focusing on the impacts of climate change on other felid species have scientifically identified their vulnerability to climate change and associated life history traits and established spatial conservation priorities under future climate scenarios (Zanin, Palomares & Albernaz, 2021; Baral et al., 2023). Research investigating the impact of climate change on predators such as clouded leopard and their suitable habitat under climate change scenarios is crucial for managing, expanding habitats, and sustainable conservation of these species. Little is known about the diet and feeding patterns of the clouded leopard (Chiang & Allen, 2017), which accounts for only 1.6% of publications on free-ranging leopards. Studies focusing on dietary composition and prey preference will aid in understanding the feeding ecology and niche partitioning among sympatric predators (Hart, Katemb & Punga, 1996). Previous studies on the feeding ecology of the bengal tiger (*P. tigris tigris*) and snow leopard (*P. uncia*) have predicted the population dynamics of their prey and future ecological shifts, which has greatly assisted park officials in conservation planning (Pun et al., 2022; Shrestha, Aihartza & Kindlmann, 2018). While scattered studies have documented the clouded leopard’s diverse diet, there remains a need for more geographically representative research on prey selection to better inform habitat suitability models and conservation planning. We recommend continued investigation into feeding ecology, including prey preferences and diet composition, particularly in understudied parts of the species’ range.

### Research approach

Both invasive and non-invasive methods are extensively employed in clouded leopard research. Camera trap study is a well-established and popularly used method in wildlife research (Kays et al., 2020). Among the non-invasive techniques, camera trap deployment stands out, particularly for estimating abundance, occurrence, activity patterns, distribution, and population density (Wilting et al., 2006; Brodie & Giordano, 2012; Wilting et al., 2012; Borah et al., 2013; Razak et al., 2019). This method is deemed optimal due to the cryptic behavior of clouded leopard, and recent advancements have further bolstered its effectiveness. Another technique, scat analysis, is commonly employed, especially for analyzing the species’ dietary habits (Swinhoe, 1862; Hazarika, 1996; Grassman et al., 2005; Rasphone et al., 2022). Clouded leopard habitat use is highly influenced by the availability of small and medium prey species, underscoring the potential conservation importance of prey species that can be identified through scat analysis (Mohamad et al., 2015). Despite its elusive behavior, direct observations have been used to assess dietary habits (Grassman et al., 2005). In the clouded leopard study, telemetry is adopted to understand its ecology and behavior. More specifically, GPS-based telemetry has been crucial in assessing animal movement and spatial usage (Mueller et al., 2011) despite its short-term effects on installed animals (Stabach et al., 2020). As an invasive approach, radio collar tracking has been used in free-ranging clouded leopard, revealing their home ranges and movement patterns (Dinerstein & Mehta, 1989; Austin, 2002; Grassman et al., 2005). Genomics approaches are crucial in comprehending fundamental aspects of wildlife biology, encompassing disease and population dynamics. They also guide conservation and management strategies aimed at wildlife populations and their habitats (Hohenlohe, Funk & Rajora, 2021). Genetic sampling from live clouded leopard has been employed for genetic studies and distinguishing between subspecies (Wang et al., 1995; Buckley-Beason et al., 2006). Moreover, diverse research approaches were identified during the review. For instance, Ghimirey & Acharya (2020) utilized secondary data from reports and newspapers to assess the illegal trade of clouded leopard. Secondary information proves vital, especially for species like the clouded leopard, where primary data is challenging to obtain. Similarly, research based on species sign survey and modeling is used to study the species occurrence and predict potential habitat, determining environmental factors affecting distribution and range limits, and evaluating coverage in existing protected areas (Shrestha et al., 2021). While various diseases have been reported, such as double infection (Zahedi et al., 1986), research on diagnosis and treatment approaches remains limited. However, research on disease studies is crucial, as it aims to diagnose diseases and support species treatment (Kophamel et al., 2022).

The distribution of research types indicates that clouded leopard research is an actively growing field. Researchers are still expanding on the knowledge base of this species through original research and using short communications and status updates to show ongoing research progress. Limited literature on photographic evidence of the species in the wild is corroborated by the fact that it is an elusive species. However, the limited number of review papers suggests that, while significant primary research is being conducted, there is a need for more efforts to consolidate information to build an understanding of clouded leopard ecology and conservation.

### Threats addressed by each publication

Clouded leopards face imminent uncertainty due to several threats, many escalating due to their interplay. Like other species of wild cats, clouded leopard faces significant challenges due to habitat loss, fragmentation, poaching, and illegal trade. Consequently, the population of clouded leopard has declined by at least 45% across their range over the past 21 years, indicating an annual decline of 1.9% (Gray et al., 2021). Our study has discovered clear research focus on these threats to clouded leopard.

Habitat loss and fragmentation are principal threats to species survival and long-term conservation outcomes globally, leading to species extinction (Gu, Heikkilä & Hanski, 2002). When considered by threat type, the highest proportion of papers included in our study focused on habitat loss and fragmentation. It is regarded as a primary threat to clouded leopards as they are strongly associated with primary tropical forests, in regions like Southeast Asia, where deforestation occurs rapidly (Grassman et al., 2005). It removes, reduces, and divides areas of key habitat and increases the interface between people and wildlife, confining the wildlife in insular refugia (Silwal et al., 2017) and exposing elusive fauna like clouded leopard to human presence. However, a study by Tan et al. (2017) in the Peninsula of Malaysia found that clouded leopards occurred more in heterogeneous landscapes, but this needs further research. Moreover, the impact of other factors like infrastructure development (Mathai et al., 2017; Kaszta et al., 2020), and climate change on habitat loss and fragmentation is well documented in other wildlife species but less explored in the case of clouded leopards.

Illegal trade poses one of the fastest and most destructive threats to many wild felid populations, including clouded leopard, as seen with other species like tigers, when it stems from poaching for commercial purposes (Macdonald et al., 2013). The importance of this threat to clouded leopards has also been reflected in different scientific publications explored by this study. Trade in clouded leopard has been internationally regulated since 1975 under the CITES. Clouded leopard is listed in Appendix I of CITES, which permits trade in specimens of this species only under exceptional circumstances. National laws also regulate and protect this species (Gray et al., 2021). Clouded leopard body parts continue to be trafficked, with seizures and trade surveys suggesting that indiscriminate hunting is likely to contribute to population declines (Ghimirey et al., 2013).

Although direct evidence is sparse, parasites and viruses may be overlooked threats to clouded leopards. A Malaysian individual was diagnosed with co-infection by *Brugia pahangi* and *Dirofilaria immitis* (Zahedi et al., 1986), parasites known in other felids to cause anemia, cardiovascular damage, and reduced survival. Canine distemper virus (CDV) has more recently been documented through molecular screening in clouded leopards and other wild felids (Kadam et al., 2022). The CDV has caused high mortality in tigers, leopards, and lions, indicating comparable risks for clouded leopards. Collectively, these findings suggest that parasitic and viral infections may impose substantial fitness and survival costs.

### Recommendations from Literature

A significant number of studies recommended more research and monitoring of clouded leopards. Research and monitoring efforts regarding clouded leopard have been noticeably lacking in publications, indicating a scarcity of studies on this species. This lack of attention to research is unsurprising, given the limited investigations conducted into the species. However, the study of clouded leopard is also impeded by a paucity of data, which could result from various factors. Firstly, the illegal activities surrounding this species may lead to a dearth of formal or informal information (Barber-Meyer, 2010). Secondly, the criminal element and associated personal safety risks may serve as significant deterrents to research (Wyler & Sheikh, 2009). Thirdly, researchers may opt, or be legally obligated, to withhold data to facilitate arrests and subsequent prosecutions (D’Cruze & Macdonald, 2015). While clouded leopard is protected in most countries across their range (Gray et al., 2021), unregulated hunting for pelts and habitat fragmentation contribute significantly to population declines (Gray et al., 2021). Nonetheless, clouded leopard still demands significant attention for research and monitoring in their habitats.

Further studies are needed to enhance our understanding of clouded leopard’s behavior and ecology and provide a stronger basis for conservation and management decisions (Razak et al., 2019). Extensive camera trapping at the landscape level has been recommended as crucial for documenting clouded leopard distribution. For instance, clouded leopard has been documented in the Terai Arc Landscape, but no extensive population study has been carried out (Shafi, Maurya & Gupta, 2019). These targeted surveys will also contribute to understanding interactions between clouded leopard, larger carnivores like tigers and leopards, and small wild cats (Poudel et al., 2019). Studying clouded leopard alongside other large felids will help clarify their predatory niches and ecological roles (Rasphone et al., 2022). Long-term research is most critical for assessing population viability, connectivity, and the effects of prey availability, habitat heterogeneity, and poaching on population dynamics (Petersen, Savini & Ngoprasert, 2020). Moreover, detailed investigations into the clouded leopard’s prey base are required to inform effective conservation strategies (Shrestha et al., 2021). These studies should be expanded across different seasons and ecosystems. Genetic research is another priority. Future studies should examine genetic differences between the different subspecies to understand evolutionary divergence, population health (Bursell et al., 2022), and interventions required for conservation.

Our review revealed that a significant portion of articles identified the need for better conservation and policies as a priority. The species’ elusive nature and the limited knowledge of its distribution and population size emphasize the need for greater effort for conservation (Wilting et al., 2006). Reintroduction efforts should be proposed considering the recovery of prey populations and habitat. Nonetheless, such initiatives should require careful genetic and ecological planning (Chiang et al., 2015). Improved genetic management and captive breeding plans and programs must be implemented to enhance species conservation in captivity (Sethi, Chauhan & Singh, 2023). Some studies have highlighted issues regarding the social structure, well-being, and reproductive health of clouded leopards in captivity. However, refined management strategies for captive breeding can address these issues, for instance, through reduced hand-rearing as suggested by Hampson & Schwitzer (2016). A holistic conservation approach might be to address widespread prey depletion, combat poaching, and ensure sustainable funding (Wolf & Ripple, 2016). Clouded leopard conservation also requires stronger policy enforcement against illegal trade, with strategic monitoring of trade routes and trends to enhance law enforcement (Borah et al., 2010; Ghimirey & Acharya, 2020).

Among the articles analyzed, habitat management was commonly recommended for clouded leopards. It is a specialized approach, given the diverse habitats the clouded leopard inhabits, including primary evergreen forests, secondary logged forests, coastal hardwood forests, coniferous forests, and grasslands (Rabinowitz, Andau & Chai, 1987; Rabinowitz, 1988; Santiapillai & Ashby, 1988; Sunquist & Sunquist, 2002). Given that clouded leopard populations also reside outside fully protected reserves, sustainable management of commercially used forests is crucial for their conservation. Limiting and regulating resource extraction in these areas can help maintain viable populations, as logging activities have contributed to population declines (Wilting et al., 2006). Additionally, informed land use planning can help maintain forest remnants and linkages critical for species persistence in increasingly fragmented landscapes (Tan et al., 2017). To prevent further habitat fragmentation, regional development planning should prioritize unprotected core habitats while also considering habitat restoration strategies (Macdonald et al., 2019). Thus, holistic species conservation requires strengthening protected area networks and securing connectivity corridors (Petersen, Savini & Ngoprasert, 2020). Lastly, targeted habitat restoration efforts in areas where clouded leopards still exist within protected landscapes are necessary to ensure the long-term survival of this species (Ma et al., 2022).

Papers recommending conservation education and awareness were very few. They were focused on assessing public perceptions and identifying anthropogenic threats to clouded leopard. They indicate that local government officials should be sensitized to environmentally friendly practices in regions where infrastructure development takes precedence over biodiversity conservation (Ghimirey & Acharya, 2018). Additionally, targeted awareness campaigns can play a crucial role in gaining local support in areas where clouded leopard reintroductions are being considered. Such campaigns can emphasize the economic benefits of conservation, such as increased tourism revenue for local communities (Greenspan et al., 2020). Furthermore, knowledge-based educational initiatives directed at key demographic groups have improved public attitudes toward clouded leopards. Addressing concerns regarding their negligible impact on livestock further enhances acceptance and support for conservation efforts (Petersen et al., 2020; Greenspan et al., 2020).

## Conclusions

This systematic review provides a comprehensive overview of the current research landscape on clouded leopard, highlighting geographic distributions, publication trends, key thematic areas, research approaches, and persisting threats and recommendations. Our findings reveal that research on clouded leopard is on an upward trajectory, reflecting growing scientific interest in the species. While significant progress has been made in understanding their ecology, particularly in captive settings, a pressing need remains for more field-based studies targeting wild populations, especially in understudied regions. Our findings indicate a critical gap in laboratory-based research within a range countries, underscoring the need to prioritize such studies. Strengthening conservation efforts in this area will require dedicated investment through both domestic and international funding support.

To address the gaps identified by our study, future research should prioritize integrating modern technologies such as camera trapping alongside the citizen science approach. Camera trapping has been instrumental in studying clouded leopard, offering key insights into its ecology. Integrating it with citizen science can expand geographic coverage, while emerging technologies like eDNA, remote sensing, and machine learning can enhance species detection and ecological assessments. Research gaps persist regarding the impacts of climate change on the distribution of the species. The species have a specific ecological niche, given that robust studies focusing on the potential impacts of climate change on the species could help forecast the future habitat and support the identification of the climate change refugia. Moreover, fostering community engagement through citizen science is crucial for sustaining long-term conservation efforts, as it builds local support for protecting clouded leopard in the wild. Our study greatly emphasizes the necessity for the formulation and execution of clouded leopard action plans across range countries, as these plans can provide strategic guidance for policymakers to allocate resources towards conservation efforts and revenue-generating activities within a collaborative framework, leading to a sustainable financial model for the conservation of the species.

##  Supplemental Information

10.7717/peerj.20421/supp-1 Supplemental Information 1 PRISMA checklist

10.7717/peerj.20421/supp-2 Supplemental Information 2 Country-wise publications

10.7717/peerj.20421/supp-3 Supplemental Information 3 Dataset

## Additional Information and Declarations

### Data Availability

The following information was supplied regarding data availability: The raw data is available in the Supplemental Files.
